# The quality of care delivered to Parkinson's disease patients in the U.S. Pacific Northwest Veterans Health System

**DOI:** 10.1186/1471-2377-6-26

**Published:** 2006-07-28

**Authors:** Kari Swarztrauber, Eric Graf, Eric Cheng

**Affiliations:** 1 Providence Health System, Newberg, Oregon, USA; 2 Department of Neurology, Oregon Health and Sciences University, Portland Oregon, USA; 3 Parkinson's Disease Research Education and Clinic Center, VA Greater Los Angeles Healthcare System, Los Angeles, California, USA

## Abstract

**Background:**

Parkinson's disease (PD) is the second most common chronic neurological disorder of the elderly. Despite the fact that a comprehensive review of *general *health care in the United States showed that the quality of care delivered to patients usually falls below professional standards, there is limited data on the quality of care for patients with PD.

**Methods:**

Using the administrative database, the Pacific Northwest Veterans Health Administration (VHA) Data Warehouse, a population of PD patients with encounters from 10/1/98-12/31/04 were identified. A random sample of 350 patient charts underwent further review for diagnostic evaluation. All patients whose records revealed a physician diagnosis of definite or possible Idiopathic Parkinson's (IPD) disease (n = 150) were included in a medical chart review to evaluate adherence to five evidence-based quality of care indicators.

**Results:**

For those care indicators with good inter-rater reliability, 16.6% of care received by PD patients was adherent for annual depression screening, 23.4% of care was adherent for annual fall screening and, 67.3% of care was adherent for management of urinary incontinence. Patients receiving specialty care were more likely to be adherent with fall screening than those not receiving specialty care OR = 2.3, 95%CI = 1.2–4.2, but less likely to be adherent with management of urinary incontinence, OR = 0.3, 95%CI = 0.1–0.8. Patients receiving care outside the VA system were more likely to be adherent with depression screening OR = 2.4, 95%CI = >1.0–5.5 and fall screening OR = 2.2, 95%CI = 1.1–4.4.

**Conclusion:**

We found very low rates of adherence for annual screening for depression and falls for PD patients but reasonable adherence rates for management of urinary incontinence. Interestingly, receiving concurrent specialty care did not necessarily result in higher adherence for all care indicators suggesting some coordination and role responsibility confusion. The increased adherence in PD patients receiving care outside the VA system suggests that patients with outside care may demand better care within the VA system.

## Background

Parkinson's disease (PD), after Alzheimer's dementia, is the second most common chronic neurological disorder of the elderly [1]. Despite the fact that a comprehensive review of *general *health care in the United States showed that the quality of care delivered to patients usually falls below professional standards [2], there is limited data on the quality of care for patients with PD. One study showed that less than one-third of patients initially diagnosed with PD by a non-neurologist were eventually referred to a neurologist for care [3], even though non-neurologists may not have adequate knowledge to manage PD patients [4].

Nevertheless, there have been no large-scale efforts to comprehensively measure the quality of PD care because, in part, because the necessary tools to perform an explicit review of the processes of PD care have not been developed. The most widely used method to measure quality is based on an explicit review of the processes of medical care [5], which is frequently conducted by structured abstraction of information from the medical record or through patient interviews. Recently, a health services research team, consisting of members of the National VA Parkinson's Disease Research, Education and Clinic Center (PADRECC), developed a set of evidence-based indicators that could be used to evaluate and measure the quality of care received by patients with Parkinson's disease, as measured by medical care process [6]. These indicators describe the care recommended through expert consensus of the scientific evidence in several domains of care, including preventative, primary and specialty care.

The goal of this research study is to evaluate how closely a select number of care areas, i.e. depression and fall screening, as well as management of orthostatic hypotension, urinary incontinence and hallucinations in Parkinson's disease patients are consistent with the care recommended by the scientific evidence supporting these recommended practices.

## Methods

### Identifying patient sample/sample size calculation

Using the administrative database, the Pacific Northwest Veterans Health Administration (VHA) Data Warehouse, the population of patients in the Pacific Northwest (Alaska, Washington, Oregon, Idaho) with encounters from 10/1/98-12/31/04 with parkinsonism ICD-9 CM codes 332.0, 333.0, 332.1, 333.90 (n = 3863) or a dopaminergic medication (n = 5864), i.e. carbidopa/levodopa or a dopamine agonist were identified. Assuming a significance level of 0.05, and power of 80%, we estimated we would need approximately 200 patients with PD to have enough power to detect a 5% difference in adherence between specialty and non-specialty visits to annual care indicators with rates of 10% and above. Because approximately 60% of patients identified by these four ICD-9 codes for parkinsonism and/or a dopaminergic medication had a physician diagnosis for idiopathic parkinsonism upon chart review in a previous study [7], a random sample of 350 patient charts underwent further review for diagnostic evaluation. The two earliest and two latest movement disorder, neurology, or primary care notes within this time period were reviewed for diagnostic confirmation. All patients (n = 150) whose records revealed a physician diagnosis of definite or possible Idiopathic Parkinson's disease were included for review of the quality of care indicators. Information was also collected on diagnostic criteria for parkinsonism such as rigidity, tremor, bradykinesia, and postural instability. Supportive clinical criteria for IPD was collected, e.g. unilateral onset, excellent response to levodopa, progressive nature, persistent asymmetry, levodopa induced motor fluctuations, and response to levodopa greater than five years. Non-supportive criteria were also collected such as bilateral onset, non-response to levodopa, history of schizophrenia, strokes or head injury, early dementia, and cerebellar findings.

### Selection of care indicators

Five care indicators were chosen for use in this analysis representing a range of preventative, primary and specialty care from the original 16 PD-specific "Evidence-Based Guide to Key Care Processes in Parkinson's Disease Management" indicators that met expert consensus criteria for needing room for improvement and having high impact on patient outcomes if improved[6]. These indicators were chosen because they represent a broad range of preventive, primary and specialty care areas: annual screening for depression, annual screening for falls, management of urinary incontinence, management of orthostatic hypotension and management of hallucinations. The list of indicators including their eligibility and adherence criteria is shown in Table 1.

### Development of medical chart abstraction instrument

Each week, members of the research team (KS, EC, EG, BGV) tested the ability of the instrument to accurately extract information about the care indicators. The research team met weekly to discuss the instrument performance and to make adjustments. The instrument development team also included Health Services Researchers at the VA Greater Los Angeles Healthcare System who were involved in the development of the original indicators. Using Microsoft Access software, interface forms were created. The instrument was constructed to have multiple linked Access interface forms that seamlessly guide the medical chart reviewer through questions about the presence or absence of recorded data within each note on a note-by-note basis. Reviewers (EG, JA) are asked to record dates and clinics for all entered data. The layout of the instrument is such that it asks for the presence or absence of data elements in each note. Therefore, time period or conditional requirements for indicator eligibility and adherence are calculated by post hoc data analysis, rather than asking reviewers to assess eligibility or adherence across different notes and time periods. This methodology is objective and therefore conforms more closely to explicit judgment. This instrument was developed and tested during a six month period by the research team in collaboration with researchers at the VA Greater Los Angeles Healthcare System. The instrument was pilot tested on twenty-five patient records to assess face validity and reliability prior to use.

### Data collection

Patient records from all sites in the Pacific Northwest VHA were accessed and reviewed using the Computerized Patient Record System (CPRS). A trained medical chart reviewer with a master's degree in Public Health reviewed all patient records (EG). A second trained medical chart reviewer (JA) blinded to the results of the first reviewer evaluated 7% (n = 23) charts to assess inter-rater reliability for diagnostic accuracy and for presence of care criteria for indicators. The trained medical reviewers extracted data using an Access-based interactive instrument. All patient encounters are uploaded into the instrument from the Pacific Northwest VHA Data Warehouse database and listed on a reference table in the instrument to assist the medical chart reviewer in negotiating the encounters and ensure that notes are properly included or excluded based on the criteria. All outpatient clinic visit notes written by physicians, nurse practitioners, physician assistants, psychologists or therapists for primary care, general medicine, geriatrics, neurology, neurology specialties, emergency visits, physical, kinesiotherapy and occupational therapy, rehabilitation, and mental health were reviewed. In addition, all telephone contacts for the above providers were reviewed. No inpatient records were reviewed. However extended care and residential care notes were reviewed since this is the only mechanism by which some patients are seen by a healthcare provider in the Pacific Northwest VHA. Notation of non-VA care was also recorded. The study was approved by the Portland VA Medical Center Internal Review Board and Research & Development Committee, VA #02-3404.

### Analysis

Differences in patient characteristics and demographics were compared using chi-square and t-test analyses for patients whose diagnosis qualified them for further indicator review and for patients whose diagnosis did not qualify them for further review.

Inter-rater reliability was evaluated using the Kappa co-efficient for the qualifying diagnoses and the presence of the care criteria for each indicator for each patient. Adherence and eligibility rates and bi-variate analyses are reported in patient-years. For each patient-year, there was a designation of eligibility (yes/no) for the indicator and whether the patient received care that adhered to the quality indicator (yes/no) for each encounter. To calculate eligibility and adherence in patient years, any encounter from the first visit to a period within 364 days is considered the patient's first year, any visits within 365–729 days is the second year, etc. A patient-year is defined as having specialty care if any encounter during that period was for specialty care: geriatrics, neurology or movement disorders visit. A patient-year is defined as having non-VA care if any encounter during that period noted non-VA care. *Any *encounter during the year that qualifies the patient to be eligible or adherent results in that patient-year being assigned an adherent or eligible patient-year. Bi-variate analyses using chi square and Fisher's exact test were used to evaluate the association between adherence to indicators of care and the presence of specialty care during that year and the presence of non-VA care.

The average number of encounters per eligibility period was also calculated for patients who received specialty care and for patients who did not receive any specialty care.

## Results

### Characteristics of patients with and without a qualifying diagnosis for further PD care indicator abstraction

317/350 patients had adequate medical records allowing for determination of a patient diagnosis. Of the 317 patient records, 150 had either idiopathic Parkinson's disease or possible idiopathic Parkinson's disease. Not surprisingly, patients deemed to have PD differed from those who were not deemed to have PD on most demographic and clinical characteristics evaluated. (Table 2.) The Kappa co-efficient for inter-rater reliability for a qualifying diagnosis was 1.0, with 95% CI: 0.6–1.4.

### Patient encounters

44/150 or 29.3% of PD patients had care outside the VA mentioned in their records. 79/150 or 52.7% of PD patients had seen a specialist at some point in their care. The average number of encounters per eligibility period for fall and depression screening (per patient-year) was 5.3 for those receiving specialty care and 2.7 for those not receiving specialty care. (p < .001) The majority of visits for PD patients was for primary care and internal medicine. (Table 3.)

### Rates of adherence for quality indicators

**Figure 1:**
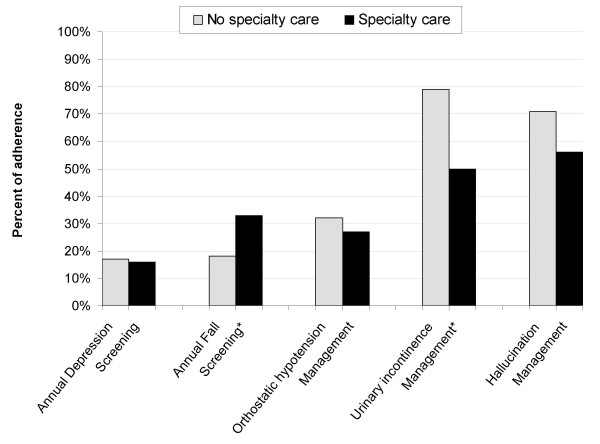
**Adherence to care by presence of concurrent specialty care**. *Chi square significant at the p < 0.05 level

**Figure 2:**
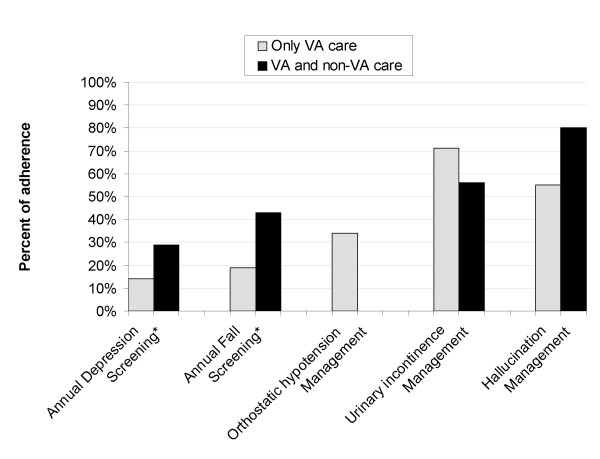
**Adherence to care indicators by VA care only vs. VA and non-VA care**. *Chi square significant at the p < 0.05 level

For annual depression screening, 16.6% of care received by PD patients was adherent. 23.4% of care was adherent for annual fall screening. 29.7% of care was adherent for management of orthostatic hypotension, 67.3% was adherent for management of urinary incontinence, and 60% was adherent for management of hallucinations. Kappa coefficients for inter-rater reliability exceeded 0.7 for recording the presence of depression, falls and urinary incontinence. The Kappa coefficient for the presence of orthostatic hypotension and hallucinations could not be calculated due to not enough instances of an assessment recorded as positive to reliably report management. Patients receiving specialty care in a given year were more likely to be adherent with fall screening than those not receiving specialty care OR = 2.3, 95%CI = 1.2–4.2, but less likely to be adherent with management of urinary incontinence, OR = 0.3, 95%CI = 0.1–0.8. (Figure 1.) Patients receiving care outside the VA system were more likely to be adherent with depression screening OR = 2.4, 95%CI = >1.0–5.5 and fall screening OR = 2.2, 95%CI = 1.1–4.4. (Figure 2.)

## Discussion

Our rates of adherence for depression screening were remarkably low as reported in the provider's encounter note and the indicator demonstrated good inter-rater reliability. In the Pacific Northwest VHA, the majority of patients are screened for depression by a primary care intake nurse but the results are not reported in encounter notes. Our study only extracted provider encounter notes. The provider must be aware of the screen results and feel the results are clinically important to make a notation in their encounter note. Thirty-five percent of the PD patients in our sample had been given an ICD-9 CM code for depression and the prevalence of depression in other PD patient populations has been shown to be 40–50% [8,9]. Therefore, it is likely that providers are either 1) not determining the screening results of their patients or 2) not recording the presence or absence of depression in their PD patients' management. Although encounter visits doubled after patients began seeing specialists, the rate of annual screening for depression did not improve. Given that depression is so prevalent in PD patients and that depression and disability are associated [10], our findings strongly suggest that providers are neither recording the presence of depression nor considering depression an important co-morbidity in PD patients. Interestingly, patients with non-VA care had nearly double the rate of depression screening recorded in the encounter note compared to those without outside care. Since we did not review outside notes, this increased rate reflects VA providers noting the presence or absence of depression in their own evaluation and management. The higher rate of depression screening in patients with outside VA care could be for several reasons. Patients with outside care may be more pro-active and demand their providers be more thorough in their management. VA providers may be also more thorough because they worry that patients with outside care may not be getting as good as care as patients without outside care.

Adherence to recommended fall screening was also very low and the indicator demonstrated good inter-rater reliability. The most common complications of patients with PD are axial features such as gait impairment and falls [11]. In a prospective study of PD patients with average duration of illness of three years, falls occurred in over two-thirds of PD patients annually with half of the PD patients experiencing more than one fall [12]. For 100 PD patients followed over two months, 20 hospitalizations occurred for falls of which three-quarters were for fractures and the rest for lacerations [13]. Finally, falls are a leading cause of nursing home admissions in a general elderly population [14]. Given the prevalence of falls and its association with poor patient outcomes, the low adherence rates for screening are concerning.

Patients who received care outside the VA had higher screening rates for falls and this may be for the same reasons as the higher depression screening rates seen in patients receiving outside care. However unlike depression screening, patients with specialty care had higher rates of adherence for annual fall screening. There are two possible reasons why patients who are seeing specialists have a higher rate of adherence to annual fall screening. It may be that patients that see specialists are more likely to have fallen and therefore report falls to their providers. Alternatively, it may be that specialists are more aware of falls as a complication of PD and are more likely to ask patients about falls. Regardless, considering that even with specialty care, adherence rates were less than a third, interventions specifically targeting fall screening should be of prime importance.

Discussion of treatment options for urinary incontinence had the highest adherence rates of any of the indicators and the indicator also demonstrated good reliability. Greater than half of patients who had symptoms of incontinence received appropriate management advice. Surprisingly, patients who received specialty care were much less likely to receive appropriate care. The high adherence rate among non-specialty providers may reflect the fact that urinary incontinence occurs with some frequency in patients without PD. Nevertheless, urinary symptoms occur in 40% of PD patients [11]. Our finding that patients who received specialty care in addition to primary care had lower rates of adherence suggests that there may be a negative outcome from the coordination of care between specialists and generalists. Both provider groups may believe that the other provider group is responsible for managing urinary incontinence.

Inter-rater reliability for the management of hallucinations could not be calculated due to not enough instances of an assessment recorded as positive to reliably report management. Of those patients who had hallucinations, greater than half were managed appropriately. The presence of hallucinations has been shown to be a stronger predictor of nursing home placement for patients with PD compared to motor and cognitive impairment [15,16]. In particular, the presence of hallucinations early in the treatment of parkinsonism is associated with increased probability of nursing home placement [17]. The low number of eligibility periods we found in this study suggests that providers may not be asking or recording the presence of hallucinations. Because of the importance of management of hallucinations for patient outcomes, future research may want to first focus on accurate screening for hallucinations.

Inter-rater reliability for the management of orthostatic hypotension also could not be calculated due to not enough instances of an assessment recorded as positive to reliably report management. Adherence was less than a third and none of the predictor variables influenced adherence.

In addition to evaluating rates of adherence to several evidence-based care indicators in patients with PD, this study had two other important findings. Our study confirmed that medical chart review is necessary to identify cases of PD. Less than half of the patient medical charts identified with administrative data had confirmed or probable PD on medical chart review. The author has shown previously that administrative data is inaccurate in identifying cases of PD for quality of care assessments [7]. This study also found that only half of the veterans with PD were seen by specialists. This is consistent with other studies of non-VA PD populations in both the U.S. and Canada that have shown PD patients have limited access to specialty care [3,18].

Our study is limited in that two of the care indicators had too few instances of an assessment recorded as positive to reliably report management and therefore caution must be taken in interpreting the results of those indicators. For the rest of our indicators and for qualifying diagnoses, inter-rater reliability was high. While blinded, both our raters were involved in the development of the abstraction instrument and therefore their familiarity with the instrument may have resulted in higher rates of reliability than might otherwise be found in raters.

Another limitation of our study is that our rates of adherence to PD care indicators may not be generalizable outside the Pacific Northwest VA system. Rates reported by the VA Greater Los Angeles Healthcare system using a similar instrument were higher than those reported by us: annual screening rates for depression of 67% and annual screening rates for falls of 52% [19]. The differences in rates between the two VHA systems may be a result of the higher prevalence of PD specialty care at the VA Greater Los Angeles Healthcare system or the fact that care was evaluated in the year 2003 at West Los Angeles VA whereas care was evaluated from 1998–2004 for the Pacific Northwest VA. Regardless, these findings suggest a large amount of geographic variability of care delivered to PD patients and require further evaluation.

The findings of this study suggest that physician professional societies or Parkinson's disease organizations might consider promoting screening for PD patients to improve adherence to care indicators. These organizations could make available a standardized set of questions about recent falls, hallucinations, symptoms of orthostatic hypotension and a self-administered depression scale that PD patients could complete prior to their clinic visits.

## Conclusion

We found very low rates of adherence to evidence-based care indicators for annual screening for depression and falls for PD patients but some reasonable adherence rates to indicators for management of urinary incontinence. Because reliability estimates could not be obtained, adherence rates to indicators for management of hallucinations and orthostatic hypotension should be interpreted with caution, but the low rates of eligibility suggests initial screening for these disorders may be warranted. Interestingly, receiving concurrent specialty care did not necessarily result in higher adherence for all care indicators suggesting some coordination and role responsibility confusion. The increased adherence in PD patients receiving care outside the VA system suggests that patients with outside care are treated differently by VA providers.

## Competing interests

The authors have no competing interests.

## Authors' contributions

KS, EG and EC approved the final manuscript. KS wrote the core manuscript. EC, EG contributed to sections specifically introduction, methods and tables. KS, EC conceptualized the study. EG, KS, EC developed and tested the quality of care instrument. EG collected data and performed the data analyses. EG, KS interpreted the data analysis.

## Pre-publication history

The pre-publication history for this paper can be accessed here:



## Figures and Tables

**Table 1:** Adherence and eligibility criteria and level of evidence for the Parkinson's disease quality of care indicators

	Defined eligibility = denominator	Defined adherence = numerator	Level of evidence*	Domain
**Urinary incontinence management**	PD patient with new urinary incontinence or worsening urinary incontinence	Documenting that discussed with patient and offered patient several treatment options	A1	Management of **non **motor complications
**Annual depression screening**	Each year in cohort for patients with PD	Screening for depression with at least note of the presence or absence	A1	Management of dementia, depression, psychosis
**Annual fall screening**	Each year in cohort for patients with PD	Medical record documents patient asked about falls at least once a year	Indirect	Management of motor complications
**Orthostatic hypotension management**	Patient with symptomatic orthostatic hypotension not responsive to behavioral modification	Prescribing midodrine or fludrocortisone	A1/C1	Management of **non **motor complications
**Hallucination management**	Patient with PD having persistent hallucinations or delirium not improved by a discontinuation or reduction of PD medications	Prescribing quetiapine or clozapine rather than prescribing contraindicated neuroleptic or no anti-psychotic at all	A1	Management of dementia, depression, psychosis

*A1 = randomized clinical trial, B1 = prospective observational study, C1 = cross-sectional or historical observational study shows benefit

**Table 2:** Demographics and characteristics of the patient records that were, and were not, deemed to have possible or definite idiopathic Parkinson's disease

	Deemed to have IPD N = 150	Deemed not to have IPD N = 167	p-value
Date of birth, mean	1929	1935	<0.0001
Age at death, mean, n	75.8, n = 10	79.6, n = 16	0.30
Period of Service, no. (%)			0.01
WWII	(50.3)	(30.4)	
Vietnam	(22.6)	(35.6)	
Other	(27.0)	(34.0)	
Percent service connected, mean	57.3	62.7	<0.0001
Gender, no. (%)			0.09
Male	(96.9)	(92.7)	

ICD-9 CM codes, no. (%)			
332.0	(76.1)	(30.4)	<.0001
332.1	(14.4)	(0.6)	0.006
333.0	(8.8)	(4.7)	0.12
333.90	(5.0)	(10.5)	0.06
311 (depression)	(35.2)	(37.1)	0.71

Diagnoses			
IPD	113		-
Possible IPD	37		-
Medication-induced parkinsonism		17	-
Parkinsonism		10	-
Non-parkinsonism movement disorder		93	-
Other neurological disorder		47	-
Clinical characteristics			
Postural instability	26 (17.3)	4 (2.4)	<0.0001
Bradykinesia	51 (34.0)	5 (3.0)	<0.0001
Rigidity	46 (30.7)	8 (4.8)	<0.0001
Resting tremor	61 (40.7)	11 (6.6)	<0.0001
Gait difficulty	47 (31.3)	6 (3.6)	<0.0001

**Table 3:** Type of encounter visits for patients deemed to have idiopathic Parkinson's disease

Clinic	Number of visits	Percent of all visits
Primary Care/Medicine	384	46.8
Neurology	240	29.2
Mental Health/Psychiatry	78	9.5
PADRECC*	52	6.3
Geriatrics	20	2.4
Physical therapy	27	3.3
Kinesiotherapy	16	2.0
Occupational therapy	4	0.5
Total	821	100

*Parkinson's Disease Research Education and Clinical Center
